# Time-Sequencing of the Neutrophil-to-Lymphocyte Ratio to Predict Prognosis of Triple-Negative Breast Cancer

**DOI:** 10.3390/cancers13143472

**Published:** 2021-07-11

**Authors:** Joo-Heung Kim, Nak-Hoon Son, Jun-Sang Lee, Ji-Eun Mun, Jee-Ye Kim, Hyung-Seok Park, Seho Park, Seung-Il Kim, Byeong-Woo Park

**Affiliations:** 1 Department of Surgery, Yongin Severance Hospital, Yonsei University College of Medicine, Yongin 16995, Korea; PIANAC@yuhs.ac (J.-H.K.); JSL72@yuhs.ac (J.-S.L.); 2 Division of Biostatistics, Yongin Severance Hospital, Yonsei University College of Medicine, Yongin 16995, Korea; NHSON@yuhs.ac; 3 Data Science Team (Biostatistician), Center for Digital Health, Yongin Severance Hospital, Yonsei University College of Medicine, Yongin 16995, Korea; MUNJE622@yuhs.ac; 4 Department of Surgery, Yonsei University College of Medicine, Seoul 03722, Korea; JEEYE0531@yuhs.ac (J.-Y.K.); IMGENIUS@yuhs.ac (H.-S.P.); PSH1025@yuhs.ac (S.P.); BWPARK@yuhs.ac (B.-W.P.)

**Keywords:** triple-negative breast cancer, neutrophil-to-lymphocyte ratio, prognostic biomarker, adjuvant therapy, time-serial analysis

## Abstract

**Simple Summary:**

Although the outcomes of breast cancer have improved, triple-negative breast cancer (TNBC) still has a poor prognosis. Since the prognosis of TNBC varies, identifying subgroups with particularly poor prognosis is important. The neutrophil-to-lymphocyte ratio (NLR), a systemic biomarker, is a prognostic factor for breast cancer. Since some studies report negative results on the effect of NLR and most analyze only the preoperative baseline NLR, it is difficult to apply the results in actual clinical treatment. Our study showed that the change in the NLR from before surgery to 1 year after surgery gradually increased in the disease progression group and decreased in the no evidence of disease group. Therefore, in addition to standard treatment, immunotherapy, additional chemotherapy, or clinical trials should be considered for TNBC patients who show an increased NLR during adjuvant treatment.

**Abstract:**

Since triple-negative breast cancers (TNBCs) have varying prognoses, it is important to identify subgroups with particularly poor prognosis. The aim of this study was to assess whether changes in the neutrophil-to-lymphocyte ratio (NLR) during the treatment process were associated with poor prognosis in TNBC patients. This study included 600 TNBC patients who underwent surgery from January 2005 to December 2016. The associations of the NLR and clinicopathologic factors with breast cancer recurrence and survival in patients who underwent both definitive local treatment (total mastectomy or breast-conserving surgery with radiotherapy) and systemic chemotherapy were analyzed. The NLRs at four time points (before surgery, before chemotherapy, before radiotherapy, and 1 year after surgery) were assessed. The univariate analysis showed that changes in the NLR before the start of radiotherapy (odds ratio: 1.115, confidence interval: 1.011–1.229) and 1 year after surgery (odds ratio: 1.196, confidence interval: 1.057–1.354) significantly increased the risk of recurrence or death. In multivariate analysis, T stage, N stage, and changes in the NLR were significant factors. A time-sequenced NLR may reflect the prognosis of TNBC patients. A poor prognosis is expected in patients whose NLR increases during treatment compared to the preoperative NLR, and additional treatment is needed.

## 1. Introduction 

Breast cancer is the most common cancer in women worldwide and is the leading cause of cancer-related death in women [1]. Increased research on breast cancer has improved diagnosis and treatment methods, and the prognosis of breast cancer has gradually improved [2]. However, triple-negative breast cancer (TNBC), which accounts for about 10–20% of all breast cancers, has the poorest prognosis among the breast cancer subtypes because of the absence of therapeutic targets such as hormone receptors and human epidermal growth factor receptor 2 (HER2) [3,4,5]. Since TNBCs are not a homogeneous group and have varying prognoses, it is important to identify subgroups of TNBC patients with particularly poor prognosis [6,7].

The neutrophil-to-lymphocyte ratio (NLR), absolute lymphocyte count (ALC), platelet-to-lymphocyte ratio (PLR), and systemic immune-inflammation index (SII) are systemic biomarkers that have been reported to serve as prognostic factors in breast cancer [8,9,10,11,12]. The NLR has a significant relationship to the prognosis of breast cancer, and a high NLR is associated with low disease-free survival and overall survival [13,14]. Although subgroup analyses have shown that the NLR reflects prognosis, particularly in TNBC, some studies reported different results according to cancer severity or administration of neoadjuvant chemotherapy (NeoCTx) [13,15,16]. Therefore, further research is needed. 

Previous studies examining the NLR as a prognostic factor for breast cancer used the preoperative or pre-NeoCTx NLR. Therefore, these studies do not reflect changes that occur during treatment, and it is difficult to use the NLR to determine the course of treatment as it is applied in practice [13,14,15,16]. Time-serial analysis using the NLR before, during, and after treatment may allow better prognostic prediction after the completion of treatment by reflecting reactions during the treatment process.

In this study, time-serial analysis of the NLR was performed by assessing the NLR at several time points in TNBC patients who completed postoperative chemotherapy (CTx). Data on the NLR were collected at diagnosis and after surgery, CTx, and radiation therapy (RTx). The aim of the study was to determine whether changes in the NLR during the treatment process in TNBC patients who received standard treatment could predict poor prognosis. 

## 2. Materials and Methods

### 2.1. Study Population

In this study, patients with TNBC who underwent surgery in the Severance Hospital of Yonsei University College of Medicine from January 2005 to December 2016 were included. All patients were treated with definitive local treatment (total mastectomy or breast-conserving surgery followed by RTx) and systemic CTx. Under the guidelines of the American Society of Clinical Oncology/College of American Pathologists (ASCO/CAP), TNBC was defined as breast cancer without expression of hormone receptors or HER2 [17]. HER2 immunostaining was scored from 0 to 3+, and in situ hybridization (INFORM HER2 Dual ISH DNA Probe Cocktail Assay, Roche Diagnostics, Rotkreuz, Switzerland) was performed in cases with 2+ (equivocal) immunostaining. HER2 gene amplification was defined as a HER2/chromosome 17 copy number ratio ≥2.0 according to the ASCO/CAP guidelines [18]. HER2 negativity was defined as scores of 0 or 1+ or HER2 2+ without gene amplification.

The exclusion criteria were as follows: (1) age <18 years; (2) Stage 4 disease at diagnosis; (3) NeoCTx; (4) bilateral breast cancer; (5) double primary cancer; (6) participation in clinical trials; (7) hematologic disease; (8) failure to complete planned adjuvant therapy (incomplete treatment); (9) insufficient data on when CTx and RTx were performed (follow-up loss); and (10) NLR data could not be identified (insufficient data).

### 2.2. Data Collection

In the prospectively collected Severance Breast Cancer Registry, the target patients’ demographic information, including age, sex, weight, and height, was collected, and the methods of surgery were classified as total mastectomy or partial mastectomy based on the range of resection of the breast. Patients were monitored until September 2019, and those who experienced recurrence or died of breast cancer were identified as the disease progression group. Other patients were classified as the no evidence of disease (NED) group. The follow-up and observations included regular physical examinations at 6-month or 1-year intervals, blood tests, including tumor markers, and imaging tests where the patient complained of discomfort, including that in the breast.

This study was approved by the Institutional Review Board of Severance Hospital of Yonsei University and the Institutional Review Board of Yongin Severance Hospital of Yonsei University (IRB# 2021-0071-001, approved on 15 April 2021), and all researchers conducted research in compliance with Good Clinical Practice guidelines and the Declaration of Helsinki. Informed consent was obtained from all patients involved in the study.

### 2.3. Sequential NLR Measurements

NLR data were collected from all patients at four time points. Time point 1 was the baseline, which was prior to surgery. Time point 2 was after surgery and before CTx. Time point 3 was between CTx and RTx. For patients who did not receive RTx, Time 3 was defined as the time point 4 weeks after the last CTx administration. Time point 4 was 1 year after surgery, which corresponded to approximately 6 months after RTx. If there were multiple NLR values between the individual time points, the most recent value was used to minimize the effectiveness of the intervention. Other inflammatory biomarkers, including the ALC, PLR, and SII, were also assessed at the same time points.

### 2.4. Statistical Analysis

The NLR data obtained at the four time points were used for statistical analysis as repeated measurement data. The change in the NLR was evaluated using a linear mixed model with random intercept and slope as implemented in the MIXED procedure of SAS (Version 9.4; SAS Institute, Cary, NC, USA) with a restricted maximum likelihood estimation. This analysis uses the observed data from each patient with no imputation for missing data. T stage, N stage, histologic grade, CTx regimen, and the NLR by time interaction were fixed. In this model, we analyzed the interaction between the group and time after adjusting for baseline T stage, N stage, histologic grade, and CTx regimen, which allowed us to examine differential changes in the NLR over time in the two groups. Additionally, we performed post hoc analyses to estimate the time points at which the effects differed between the two groups. In the post hoc analysis, the least square means of the two groups were estimated by the MIXED procedure at each time point and compared by independent two-sample t-test or Wilcoxon rank sum test. Categorical variables were summarized using frequencies and percentages and compared using the Chi-square test or Fisher’s exact test. Continuous variables were summarized using means and standard deviations and compared using the independent t-test or Wilcoxon rank sum test. The Shapiro–Wilk test was used to test for the normality of the distribution. These statistical tests were two-sided, and *p* values <0.05 were considered statistically significant.

## 3. Results

### 3.1. Clinicopathologic Characteristics

There were 827 patients diagnosed with TNBC during the study period who did not receive NeoCTx. Among them, 600 underwent surgery and adjuvant CTx and met the conditions of the study. These patients were divided into the NED group and the disease progression group (the group in which recurrence or death from breast cancer occurred). 

All patients completed systemic CTx after definitive local treatment, total mastectomy, or breast-conserving surgery. There were differences between the two groups in the baseline NLR, T stage, and N stage (Table 1). There were significantly more advanced cancers in the disease progression group than in the NED group, and the average baseline NLR was lower in the disease progression group than in the NED group. There was no difference in age, histologic grade, or proportion of patients administered granulocyte colony-stimulating factor (G-CSF) between the two groups. A comparison of the NLR between the two groups at the four time points showed that the baseline NLR (Time point 1) in the disease progression group was significantly lower than that in the NED group, but the NLR 1 year after surgery (Time point 4) was significantly higher in the disease progression group than in the NED group (Table 2). Similarly, the PLR, ALC, and SII also showed significant differences between the two groups 1 year after surgery, but the deviations were relatively large and inconsistent.

### 3.2. Linear Mixed Model Analysis

The linear mixed model analysis showed that changes in the NLR after CTx (Time point 3) and 1 year after surgery (Time point 4) significantly increased the risk of recurrence or death in the univariate analysis. After adjusting for T stage, N stage, histologic grade, and CTx regimen, the NLRs at Time points 2, 3, and 4 were still significant in the multivariate analysis (Table 3). T stage and N stage were statistically significant in both the univariate analysis and the multivariate analysis, but there was no significant relationship between histologic grade and recurrence or death. The CTx regimen was significant in univariate analysis but not in multivariate analysis. After adjusting for the LVI, the NLR was still significant (Supplementary Table S1).

### 3.3. Time-Sequence Plot

**Figure 1:**
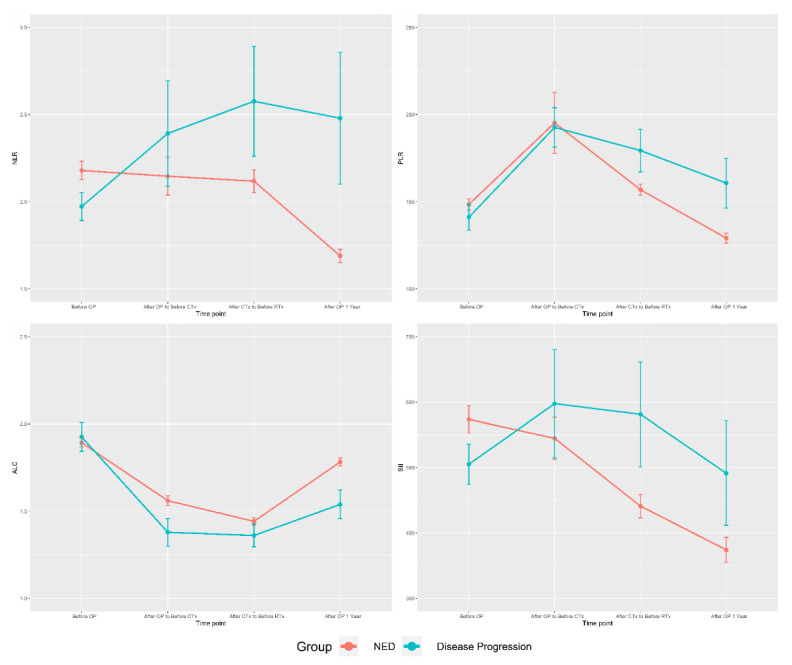
The inflammatory biomarkers according to disease progression. NLR: neutrophil-to-lymphocyte ratio; PLR: platelet-to-lymphocyte ratio; ALC: absolute lymphocyte count; SII: the systemic immune-inflammation index; CTx: chemotherapy; RTx: radiation therapy; NED: no evidence of disease.

The average inflammatory biomarkers values over time in the two groups were graphed (Figure 1). In the NED group, the NLR was maintained or decreased over time. Although the baseline NLR in the disease progression group was lower than that in the NED group, it increased with surgery, CTx, and RTx and was significantly higher than that of the NED group after CTX. The other inflammatory biomarkers had large deviations and low statistical consistency. Graphs comparing the NED group and the deceased patient group for each biomarker also showed a similar pattern (Supplementary Figure S1).

## 4. Discussion

TNBC is the least common subtype of breast cancer, but it has the worst prognosis [3,4,5]. The prognosis of TNBC patients who receive standard treatment varies; thus, these patients need to be stratified [6,7,19]. For patients expected to have a worse prognosis, additional treatments to improve the prognosis should be considered. In this study, we examined changes in the NLR in TNBC patients with poor prognosis who underwent surgical and postoperative treatment to determine whether it could be used as a biomarker to identify patients requiring additional treatment after standard treatment. Using time-sequencing of the NLR, we confirmed that the NLR increased in the disease progression group during standard treatment, whereas it was stable or decreased in the NED group. Therefore, if the NLR increases during the standard treatment of TNBC, additional treatment should be considered.

Inflammatory blood markers such as the NLR are attractive prognostic factors because they are calculated solely from peripheral blood samples. This allows easier methods of sampling and repetitive measurements than other tests. Other previous studies have reported that hematologic factors, including the NLR, are associated with prognosis in breast cancer [8,16,20,21]. A recent study also reported local immunity factors and systemic immunity factors as biomarkers of breast cancer [20,22,23]. However, some studies have reported that only the ALC and PLR are relevant to prognosis, but the NLR is not [10,24,25], and there are limited reports on the proper timing for blood sampling to evaluate the prognostic impact of the NLR [21,26].

This study showed that stepwise changes in the NLR during treatment of TNBC, including surgery, were related to prognosis, even when adjusting for stage, histologic grade and CTx regimen (Table 3). We made this group of patients as homogeneous as possible to determine the effect of the NLR on the prognosis of TNBC. Data were collected by assessing the NLR before surgery (Time point 1), after surgery (Time point 2), after CTx (Time point 3), and after RTx (Time point 4), and in cases in which the NLR was assessed multiple times within these windows, the last value was selected to minimize the short-term effects of each treatment. For example, if multiple NLR values were obtained between surgery and the beginning of CTx, we used the last NLR obtained before the administration of the first cycle of CTx. After dividing patients into NED and disease progression groups, we observed a trend of maintenance and decrease in the NLR in the NED group and a pattern of steady increase in the NLR in the disease progression group (Figure 1). The NLR’s effects on the prognosis of TNBC also showed significant results in correcting T stage, N stage, histologic grade, and CTx regimen (Table 3). The effect of NLR was also statistically significant in a further analysis of only patients with lymphovascular invasion information (Supplementary Table S1).

These results are similar to those of Patel et al., who found a significant association between an increase in the NLR and shorter survival after CTx and RTx in TNBC patients [21]. Additionally, Moldoveanu et al. confirmed a significant increase in the NLR before recurrence or death in patients with TNBC [26]. However, the study by Patel et al. included patients who did not receive CTx, and the impact of RTx on the NLR was not corrected because all patients had received RTx [21]. Therefore, it is difficult to determine the need for additional treatment based on the NLR after treatment for TNBC patients who received CTx. In addition, Moldoveanu et al. retrospectively examined the NLR in patients who experienced recurrence or died, so we cannot use their results as the basis for planning additional treatment following standard treatment by measuring the NLR after treatment, as in this study [26]. When the NLR value rises during adjuvant treatment, it is expected that the effective function of the immune system is inhibited and the probability of metastasis or recurrence of breast cancer increases [27,28].

This study aimed to not only examine the NLR at a specific point, such as before surgery or after the completion of treatment, but also to analyze the NLR over the entire treatment period to identify changes in the NLR. As we are classifying patient prognosis based on the change in the NLR and not a specific absolute NLR value, this can help predict patient prognosis during treatment and follow-up. Therefore, patients with an increase in the NLR in the course of standard treatment of TNBC may be considered for additional treatments such as immunotherapy or additional chemotherapy. 

The limitations of this study include that it is a retrospective study with a relatively small number of patients. However, the patients included in this study were meaningful in that they were relatively homogeneous patients who received CTx, a standard treatment among those diagnosed with TNBC in a single institution. Future studies validating our results and translational studies including a large group of patients to identify the mechanism by which the NLR reflects patient prognosis are needed.

Another limitation is that CTx and RTx can affect the NLR. CTx and RTx can cause bone marrow suppression effects. During CTx, G-CSF can be administered to treat neutropenia. However, neutrophils can remain in the blood for hours to days, whereas the half-life of G-CSF is 3.5 h for first-generation G-CSF and 42 h for second-generation G-CSF, which means that it is expected to take several days for G-CSF to affect hematologic factors [29,30,31]. In this study, the latest value of the NLR after treatment was used to minimize the impact of CTx and RTx on the NLR. For the Time 4 NLR, we assessed the NLR at least 12 months after surgery, which was usually 6 months after the end of RTx, to minimize the effect of RTx.

## 5. Conclusions

This study was conducted to assess the NLR as a systemic biomarker to select TNBC patients with poor prognosis after adjuvant CTx and surgery. We confirmed that changes in the NLR can reflect the prognosis of TNBC patients. The change in the NLR from before surgery to a year later gradually increased in the disease progression group, whereas the NLR showed a gradual decrease in the NED group. Therefore, in addition to standard treatment, immunotherapy, additional chemotherapy, or clinical trials should be considered for TNBC patients who show an increased NLR during standard treatment, including surgery, CTx, and RTx. 

## Figures and Tables

**Table 1:** Baseline characteristics.

Characteristic	Total	NED Group	Disease Progression Group	*p*-Value
Number of patients	600 (100.00%)	545 (90.83%)	55 (9.17%)	
Age (years)	49.88 ± 10.71	50.06 ± 10.50	48.15 ± 12.56	0.2070
Preoperative NLR	2.16 ± 1.20	2.18 ± 1.24	1.97 ± 0.59	0.0349
Preoperative PLR	147.68 ± 73.12	148.33 ± 74.75	141.13 ± 54.26	0.4906
Preoperative ALC (1000 cells/mm^3^)	1.90 ± 0.57	1.90 ± 0.57	1.93 ± 0.61	0.7215
Preoperative SII	567.01 ± 467.66	573.15 ± 484.86	505.17 ± 225.19	0.5626 ^a^
T stage				0.0019
T1	362 (60.33%)	341 (62.57%)	21 (38.18%)	
T2	226 (37.67%)	194 (35.60%)	32 (58.18%)	
T3	12 (2.00%)	10 (1.83%)	2 (3.64%)	
N stage				<0.0001
N0	506 (84.33%)	470 (86.24%)	36 (65.45%)	
N1	76 (12.67%)	64 (11.74%)	12 (21.82%)	
N2	10 (1.67%)	7 (1.28%)	3 (5.45%)	
N3	8 (1.33%)	4 (0.73%)	4 (7.27%)	
Histologic grade				0.6016
Low (Grade 1)	147 (24.58%)	134 (24.68%)	13 (23.64%)	
High (Grade 2–3)	425 (71.07%)	384 (70.72%)	41 (74.55%)	
Unknown	26 (4.35%)	25 (4.60%)	1 (1.82%)	
CTx regimen				0.0139
Non taxane-based	497 (82.83%)	458 (84.04%)	39 (70.91%)	
Taxane-based	103 (17.17%)	87 (15.96%)	16 (29.09%)	
G-CSF				0.9412
No	341 (56.83%)	310 (56.88%)	31 (56.36%)	
Yes	259 (43.17%)	235 (43.12%)	24 (43.64%)	
Ki-67, N (%) ^c^	216 (36.00%)	206 (37.80%)	10 (18.18%)	0.4564 ^b^
≤20%	47 (21.76%)	44 (21.36%)	3 (30.00%)	
>20%	169 (78.24%)	162 (78.64%)	7 (70.00%)	
LVI, N (%) ^c^	425 (70.83%)	389 (71.38%)	20 (65.45%)	0.0003 ^b^
Not identified	389 (91.53%)	363 (93.32%)	26 (72.22%)	
Present	36 (8.47%)	26 (6.68%)	10 (27.78%)	

NED: no evidence of disease; NLR: neutrophil-to-lymphocyte ratio; PLR: platelet-to-lymphocyte ratio; ALC: absolute lymphocyte count; SII: the systemic immune-inflammation index; G-CSF: granulocyte colony-stimulating factor; LVI: lymphovascular invasion; CTx: chemotherapy. Categorical variables are presented as n (%), and continuous variables are presented as mean ± standard deviation. ^a^ Wilcoxon Rank-Sum test *p*-value, ^b^ Fisher’s exact test *p*-value; ^c^ Patients with Ki-67 or LVI information

**Table 2:** Time-sequenced inflammatory biomarkers.

Time Point	Entire Cohort	NED Group	Disease Progression Group	*p*-Value
NLR				
Preoperative	2.16 ± 1.20	2.18 ± 1.24	1.97 ± 0.59	0.0349
Postoperative-before CTx	2.17 ± 2.48	2.14 ± 2.50	2.39 ± 2.25	0.4812
After CTx-before RTx	2.16 ± 1.60	2.12 ± 1.50	2.58 ± 2.34	0.1595
One year after surgery	1.76 ± 1.15	1.69 ± 0.86	2.48 ± 2.68	0.0430
PLR				
Preoperative	147.68 ± 73.12	148.33 ± 74.75	141.13 ± 54.26	0.3739
Postoperative-before CTx	194.87 ± 385.61	195.10 ± 404.00	192.67 ± 83.87	0.9071
After CTx-before RTx	158.96 ± 74.33	156.88 ± 72.26	179.39 ± 90.56	0.0791
One year after surgery	131.83 ± 69.89	129.12 ± 65.69	160.73 ± 101.18	0.0348
ALC (1000 cells/mm^3^)				
Preoperative	1.90 ± 0.57	1.90 ± 0.56	1.93 ± 0.61	0.7379
Postoperative-before CTx	1.54 ± 0.63	1.56 ± 0.63	1.38 ± 0.59	0.0405
After CTx-before RTx	1.44 ± 0.49	1.44 ± 0.48	1.36 ± 0.48	0.2335
One year after surgery	1.76 ± 0.54	1.78 ± 0.53	1.54 ± 0.58	0.0019
SII				
Preoperative	567.01 ± 467.66	573.15 ± 484.86	505.17 ± 225.19	0.5626 ^a^
Postoperative-before CTx	549.14 ± 736.87	544.17 ± 748.62	597.75 ± 613.90	0.6812 ^a^
After CTx-before RTx	453.91 ± 438.29	440.98 ± 417.80	581.37 ± 594.30	0.0577 ^a^
One year after surgery	384.11 ± 450.85	374.04 ± 437.98	491.46 ± 564.53	0.0067 ^a^

NED: no evidence of disease; NLR: neutrophil-to-lymphocyte ratio; CTx: chemotherapy; RTx: radiation therapy; PLR: platelet-to-lymphocyte ratio; ALC: absolute lymphocyte count; SII: the systemic immune-inflammation index. Data are presented as mean ± standard deviation. ^a^ Wilcoxon Rank-Sum test *p*-value.

**Table 3:** Linear mixed model analysis results.

	Unadjusted Model		Adjusted Model	
	Odds Ratio (95% CI)	*p*-Value	Odds Ratio (95% CI)	*p*-Value
NLR time point				
Preoperative	1	−	1	−
Postoperative-before CTx	1.056 (0.981, 1.137)	0.1472	1.228 (1.158, 1.301)	<0.0001
After CTx-before RTx	1.115 (1.011, 1.229)	0.0292	1.269 (1.195, 1.349)	<0.0001
One year after surgery	1.196 (1.057, 1.354)	0.0047	1.321 (1.237, 1.410)	<0.0001
CTx regimen				
Non taxane-based	1	−	1	−
Taxane-based	2.160 (1.847, 2.526)	<0.0001	0.956 (0.758, 1.206)	0.7022
T stage				
T1	1	−	1	−
T2	2.678 (2.005, 3.579)	<0.0001	2.136 (1.818, 2.508)	<0.0001
T3	3.248 (1.470, 7.174)	0.0036	1.874 (1.125, 3.122)	0.0159
N stage				
N0	1	−	1	−
N1	2.448 (1.720, 3.483)	<0.0001	2.201 (1.726, 2.806)	<0.0001
N2	5.595 (2.781, 11.258)	<0.0001	5.651 (3.764, 8.485)	<0.0001
N3	13.056 (6.384, 26.699)	<0.0001	9.175 (6.281, 13.402)	<0.0001
Histologic grade				
Low (Grade 1)	1	−	1	−
High (Grade 2–3)	1.101 (0.793, 1.528)	0.5663	0.875 (0.733, 1.046)	0.1425

CI: confidence interval; NLR: neutrophil-to-lymphocyte ratio; CTx: chemotherapy; RTx: radiation therapy.

## Data Availability

The data that support the findings of this study are not publicly available due to ethical and patient consent constraints. However, data are available upon reasonable request from the corresponding author [S.I.K.] under a collaboration and data usage agreement.

## Supplementary Materials

The following are available online at https://www.mdpi.com/article/10.3390/cancers13143472/s1, Supplementary Figure S1: Inflammatory biomarkers according to survival, Supplementary Table S1. Linear mixed model analysis results for the patients with LVI information.
