# Supplementary material for: Time-Sequencing of the Neutrophil-to-Lymphocyte Ratio to Predict Prognosis of Triple-Negative Breast Cancer
Source: Cancers (Basel). 2021 Jul 11;13(14):3472. doi: 10.3390/cancers13143472 (PMC8307555; doi:10.3390/cancers13143472)
Supplement: Supplementary file 1 [file cancers-13-03472-s001.zip › cancers-1257118-supplementary.pdf]

Supplementary Figure S1. Inflammatory biomarkers according to survival.

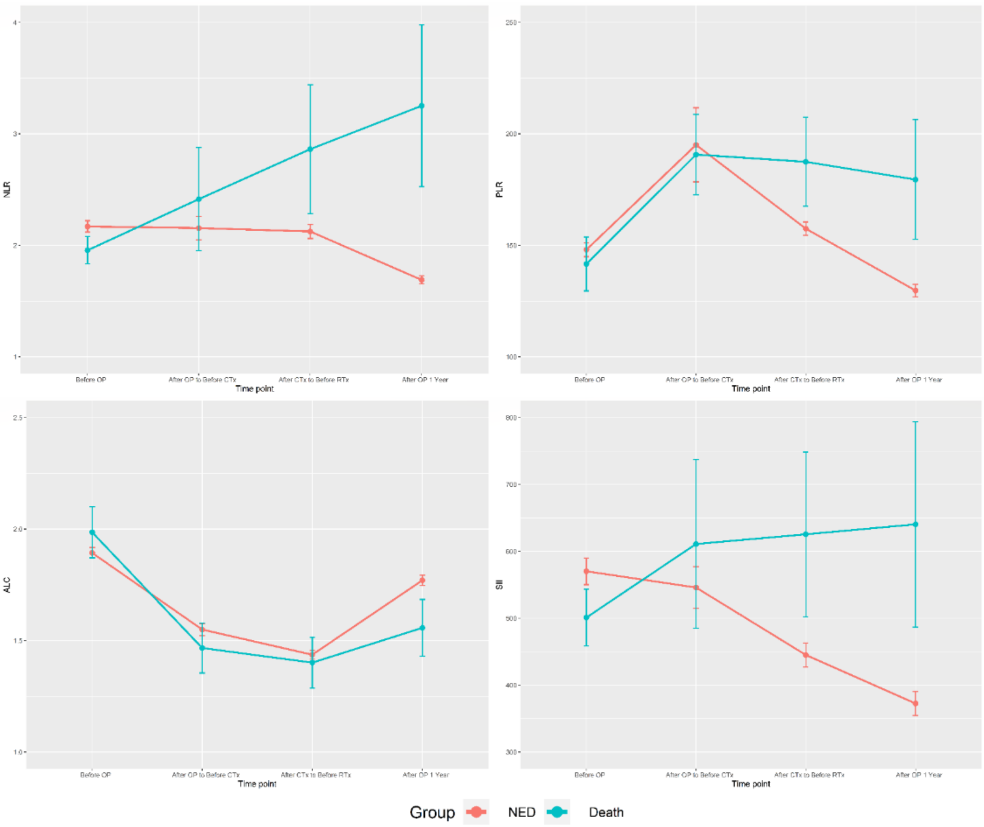

NLR: neutrophil-to-lymphocyte ratio; PLR: platelet-to-lymphocyte ratio; ALC: absolute lymphocyte count; SII: the systemic immune-inflammation index; CTx: chemotherapy; RTx: radiation therapy; NED: no evidence of disease.

Supplementary Table S1. Linear mixed model analysis results for the patients with LVI information.

	**Unadjusted model**		**Adjusted model**	
	**Odds ratio (95% CI)**	**p-value**	**Odds ratio (95% CI)**	**p-value**
**NLR time point**				
Preoperative	1	-	1	-
Postoperative-before CTx	1.233 (1.158, 1.313)	<0.0001	1.218 (1.142, 1.300)	<0.0001
After CTx-before RTx	1.270 (1.189, 1.357)	<0.0001	1.258 (1.174, 1.347)	<0.0001
One year after surgery	1.322 (1.232, 1.418)	<0.0001	1.307 (1.218, 1.402)	<0.0001
CTx regimen				
Non taxane-based	1	-	1	-
Taxane-based	1.478 (1.200, 1.821)	0.0003	0.756 (0.559, 1.023)	0.0694
Lymphovascular invasion				
Not identified	1	-	1	-
Present	5.370 (4.360, 6.614)		3.710 (2.864, 4.807)	<0.0001
**T stage**				
T1	1	-	1	-
T2	2.643 (2.227, 3.138)	<0.0001	1.960 (1.615, 2.379)	<0.0001
T3	<0.001 (<0.001, Inf)	>0.9999	<0.001 (<0.001, Inf)	>0.9999

**N stage**				
N0	1	-	1	-
N1	1.420 (1.076, 1.873)	0.0133	1.280 (0.888, 1.845)	0.1848
N2	9.583 (6.501, 14.127)	<0.0001	5.022 (3.087, 8.168)	<0.0001
N3	6.389 (4.128, 9.889)	<0.0001	3.586 (2.209, 5.821)	<0.0001
**Histologic grade**				
Low (Grade 1)	1	-	1	-
High (Grade 2~3)	2.621 (2.007, 3.423)	<0.0001	2.493 (1.867, 3.329)	<0.0001

CI: confidence interval; NLR: neutrophil-to-lymphocyte ratio; CTx: chemotherapy; RTx: radia-tion therapy; LVI: lymphovascular invasion, N=425.
